# Effective Number Theory: Counting the Identities of a Quantum State

**DOI:** 10.3390/e22111273

**Published:** 2020-11-10

**Authors:** Ivan Horváth, Robert Mendris

**Affiliations:** 1 Department of Anesthesiology and Department of Physics, University of Kentucky, Lexington, KY 40536, USA; 2 Department of Mathematical Sciences, Shawnee State University, Portsmouth, OH 45662, USA; rmendris@shawnee.edu

**Keywords:** effective number, effective measure, quantum identities, quantum uncertainty, localization, quantum computing, diversity measure, effective choices, inverse participation number

## Abstract

Quantum physics frequently involves a need to count the states, subspaces, measurement outcomes, and other elements of quantum dynamics. However, with quantum mechanics assigning probabilities to such objects, it is often desirable to work with the notion of a “total” that takes into account their varied relevance. For example, such an effective count of position states available to a lattice electron could characterize its localization properties. Similarly, the effective total of outcomes in the measurement step of a quantum computation relates to the efficiency of the quantum algorithm. Despite a broad need for effective counting, a well-founded prescription has not been formulated. Instead, the assignments that do not respect the measure-like nature of the concept, such as versions of the participation number or exponentiated entropies, are used in some areas. Here, we develop the additive theory of effective number functions (ENFs), namely functions assigning consistent totals to collections of objects endowed with probability weights. Our analysis reveals the existence of a minimal total, realized by the unique ENF, which leads to effective counting with absolute meaning. Touching upon the nature of the measure, our results may find applications not only in quantum physics, but also in other quantitative sciences.

## 1. Motivation and Overview

Among the distinctive features of quantum mechanics is that, in some regards, a quantum system acts as though it is simultaneously in multiple states of a given type. As an extreme example, a lattice Schrödinger particle in a momentum eigenstate is often said to reside at all positions, having an equal chance of being detected anywhere. However, how many of such “position identities” are effectively present in a generic state that can assign an arbitrarily varied relevance (probability) to different locations?

Variants of this counting problem appear in quantum physics quite often. One example arises in the context of Anderson localization [1] (see, e.g., [2,3] for reviews). Indeed, the Fermi-level electron inside the band of extended states (Anderson conductor) is thought of as effectively present in most of the available position states. In contrast, such an electron inside the band of localized states (Anderson insulator) only resides in a drastically reduced subset of them. Hence, a well-founded effective counting of states could be used to quantitatively describe the transition between these regimes in a novel way.

Viewing an Anderson electron from a different perspective, the effective state counting could also be used to analyze the indeterminacy (quantum uncertainty) associated with the measurement of its position. In such an approach, uncertainty would be represented by the effective number of position states the electron collapses into upon repeating the experiment: the smaller this effective number, the smaller the uncertainty. Note that the general treatment of quantum indeterminacy this way (with respect to an arbitrary basis) would be very different in nature from, e.g., the classic spectral approach [4,5].

The above types of physics analyses may usefully materialize if the generic question [Q] below can be suitably formalized and meaningfully answered. In particular, if ∣ ψ 〉 is a state from an *N*-dimensional Hilbert space and {∣ i 〉}≡{∣ i 〉∣i=1,2,…,N} its orthonormal basis (remark [6]), it is desirable to ask:

[Q] *How many states from {∣ i 〉} is the system described by ∣ ψ 〉 effectively in?*

A well-founded resolution of this “quantum identity problem” is not readily available (remark [7]). In this paper, we develop a theoretical framework (effective number theory) that gives the rationale to the following answer:

[A] *Let $P\;=\;\left(p_{1},p_{2},\ldots ,p_{N}\right)\;,\;p_{i}=\;|\;\;\langle \;i\;|\;\psi \;\rangle \;{|}^{2}$, be the probability vector assigned to quantum state ∣ ψ 〉 and basis {∣ i 〉}, and let $C\;=\;\left(c_{1},c_{2},\ldots ,c_{N}\right)\;,\;c_{i}\;=\;Np_{i}$. The system described by ∣ ψ 〉 is effectively in $N_{⋆}\left[\;|\;\psi \;\rangle ,\left\{|\;i\;\rangle \right\}\right]=N_{⋆}\left[C\right]$ states from {∣ i 〉}, where:*

(1) $$N_{⋆}\left[C\right]\;=\;\sum_{i=1}^{N}n_{⋆}\left(c_{i}\right)\;,\;n_{⋆}\left(c\right)\;=\;\min\;\left\{c,1\right\}\;for\;all\;c\in \left[0,\infty \right)$$

To arrive at [A], we start with the axiomatic definition of the effective number function (ENF) N[ ∣ ψ 〉,{∣ i 〉}] = N[C], namely a function consistently assigning the effective totals. Solving [Q] then amounts to finding such an N and using it to specify the effective number of quantum identities in all situations. The subsequent analysis shows, however, that there exists an entire continuum of ENFs. This could render each fixed choice of N too arbitrary and its individual value uninformative on its own (remark [8]). Interestingly, this is not the case because it turns out that $N_{⋆}$ is an ENF with absolute meaning. Indeed, we will prove that $N_{⋆}\left[C\right]\leq N\left[C\right]$ for all *C* and all N, making $N_{⋆}$ the unique minimum (least element) on the set of all ENFs. Having revealed that the system in state ∣ ψ 〉 has to be characterized as being simultaneously in at least $N_{⋆}\left[\;|\;\psi \;\rangle ,\left\{\;|\;i\;\rangle \right\}\right]$ states from {∣ i 〉}, this result is used in [A] as a basis for the meaningful canonical choice of ENF (remark [9]). It should be noted in this regard that a maximal ENF, whose interpretation would otherwise be on equal footing with $N_{⋆}$, does not exist (see Theorem 2).

A crucial novelty in our approach is the inclusion of additivity as a requirement for ENFs. This step is necessary since the effective number of states is an additive concept. However, a proper formulation requires some care. To that end, as well as to start invoking parallels with localization, consider the simple setting of a spinless Schrödinger particle on a finite lattice. In the position basis, its state ∣ ψ 〉 is represented by the *N*-tuple $\left(\psi \left(x_{1}\right),\ldots ,\psi \left(x_{N}\right)\right)$, with $p_{i}=\psi^{⋆}\psi \left(x_{i}\right)$ being the probability of detection at the location $x_{i}$. Denoting by C the set of all counting weight vectors $C=(c_{1},\ldots ,c_{N})$, $c_{i}\;=\;Np_{i}$, namely (remark [10]):

(2) $$C=\cup_{N}C_{N}\;,\;C_{N}\;=\;\{\;\left(c_{1},c_{2},\ldots ,c_{N}\right)\;|\;c_{i}\geq 0\;,\;\;\sum_{i=1}^{N}c_{i}=N\;\}\;$$

the additivity property for N arises as follows. Assume that the particle is restricted to a lattice of $N_{1}$ sites in a state generating the weight vector $C_{1}\;\in \;C_{N_{1}}$. Separately, let it be restricted to a non-overlapping adjacent lattice of $N_{2}$ sites and characterized by $C_{2}\;\in \;C_{N_{2}}$. With symbol ⊞ representing the concatenation operation (remark [11]), since:

(3) $$C\;=\;C_{1}⊞C_{2}\;\in \;C_{N=N_{1}+N_{2}}$$

there exists a state of the particle on the combined lattice, producing this composite *C*. Given the additivity of numbers, the sum rule for the number of available states ($N=N_{1}+N_{2}$) has to hold for its effective counterpart as well ($N\left[C\right]=N\left[C_{1}\right]+N\left[C_{2}\right]$). Consequently, the additivity property that we impose is:

(A) *Additivity:* $\;N\left[C_{1}⊞C_{2},\;N_{1}+N_{2}\right]\;=\;N\left[C_{1},N_{1}\right]\;+\;N\left[C_{2},N_{2}\right]\;,\;\forall \;C_{1},C_{2}\;$

Here, the dimensions of vector arguments were made explicit to emphasize that N[C,N] represents *N* modified by distribution *C*. Notice that $N_{⋆}$ is evidently additive and that the above reasoning does not depend on the system, state, or basis in question.

Several decades ago, Bell and Dean [12] dealt with a problem analogous to [Q] while analyzing the localization properties of vibrations in glassy silica. In particular, they asked how many atoms do these vibrations effectively spread over. Their quantifier, the participation number $N_{p}$, is given by:

(4) $$\frac{1}{N_{p}\left[C\right]}\;=\;\frac{1}{N^{2}}\sum_{i=1}^{N}c_{i}^{2}\;$$

and is still widely used in the analysis of localization. In other areas, it is common to exponentiate a suitable entropy, such as the Shannon [13] or Rényi entropies [14], and use it for analogous purposes. However, none of these quantifiers is (A)-additive. Their interpretation as effective totals is thus vague and they tend to be too arbitrary. In contrast, incorporating additivity into the definition of ENFs leads to the resolution of the quantum identity problem and suggests new possibilities both in physics and measure-related aspects of mathematics. The effective number theory, which we develop here as a tool to solve [Q], provides a theoretical starting point for such developments.

In the rest of this section, we describe the construction of ENFs and discuss the key results of effective number theory. The goal here is to provide a concise but rigorous overview, including the motivations for axiomatic properties, as well as the ramifications of deduced features. A fully mathematical treatment in the technically convenient dual form of effective complementary numbers (co-numbers) is then given in Section 2. Various generalizations of the quantum identity problem are discussed in Section 3. We then outline the use of effective numbers in quantum theory from a very broad perspective, namely as a general tool to characterize quantum states (Section 4). Concluding remarks are given in Section 5.

### 1.1. Effective Numbers

We now develop the notion of ENF as a function N = N[C], assigning an effective total to each distribution of weights C∈C over the elements of a basis. Such a construction clearly does not depend on the fact that counted objects are quantum states, and we will thus use generic terms in that regard from now on. The underlying goal is to extend the “counting measure” for a collection of distinct, but otherwise equivalent objects (natural number N∈N) to the situation when these objects acquire varied importance expressed by their counting weights (effective number N[C] ∈R). The additivity property (A) is thus a basic consistency requirement for acceptable ENFs.

Like in ordinary counting, no specific relation among individual objects is assumed. Thus, in the same way the number of balls in a bag does not change upon their reshuffle, the effective number will not change upon the permutation of counting weights. In other words, ENFs are required to be totally symmetric in their arguments, namely (remark [15]):

(S) *Symmetry:* $\;N\left(\ldots c_{i}\ldots c_{j}\ldots \right)\;=\;N\left(\ldots c_{j}\ldots c_{i}\ldots \right)\;\;,\;\;\forall \;i\neq j$

Extensions N→N[C] are by definition such that ordinary counting corresponds to all objects being equally important, and thus to a uniform distribution. More precisely:

(B1) *Boundary Condition 1:* $\;N\left(1,1,\ldots ,1\right)=N\;\;,\;\;\left(1,1,\ldots ,1\right)\in C_{N}\;\;,\;\;\forall \;N$

On the other hand, whenever all the weight is given to a single object, all others being irrelevant, the effective number is required to be one, namely:

(B2) *Boundary Condition 2:* N(N,0,…,0) = N(0,N,0,…,0) = … = N(0,…,0,N) = 1

within each $C_{N}$. Note that $\left(1,1,\ldots ,1\right)\in C_{N}$ and $\left(\ldots ,0,N,0,\ldots \right)\in C_{N}$ are the opposite extremes in the cumulation of the weight. Hence, the effective number of objects with arbitrary weights has to fall between the corresponding extremal values, namely:

(B) *Bounds:* $\;1\;\leq \;N\left[C\right]\;\leq \;N\;,\;\forall \;C\in C_{N}\;,\;\forall \;N\;$

The degree of weight cumulation plays a more detailed role in effective numbers than just determining the boundary properties. Indeed, the concept has to respect that increasing the cumulation in the distribution cannot increase the effective number. To formulate such monotonicity, consider two objects weighted by $C\;=\;\left(c_{1},c_{2}\right)\in C_{2}$ with $c_{1}\leq c_{2}$. The deformation $C\to C_{\epsilon }=\left(c_{1}-\epsilon ,c_{2}+\epsilon \right)$ leads to further cumulation in favor of the second object, and thus, $N\left[C_{\epsilon }\right]\leq N\left[C\right]$ is imposed for all $0\leq \epsilon \leq c_{1}$. In a situation with an arbitrary *N*, we require the same for each ordered pair $c_{i}\leq c_{j}$ and deformation $0\leq \epsilon \leq c_{i}$, namely (remark [16]):

(M${}^{-}$) *Monotonicity:* $\;N\left(\ldots c_{i}-\epsilon \ldots c_{j}+\epsilon \ldots \right)\;\leq \;N\left(\ldots c_{i}\ldots c_{j}\ldots \right)$

It is easy to check that (M${}^{-}$)-monotonic N attains its maximal value over $C_{N}$ at (1,1,…,1), while the minimum is at one or multiple fully cumulated vectors (…,N,…). Conditions (B1), (B2), and (B) are thus compatible with (M${}^{-}$) (remark [17]). Note that, although not an ENF, the participation number (4) satisfies (M${}^{-}$)-monotonicity.

The final requirement in the definition of ENFs is continuity. The nature of problems with admitting discontinuities can be illustrated by:

(5) $$N_{+}\left[C\right]=\sum_{i=1}^{N}n_{+}\left(c_{i}\right)\;,\;n_{+}\left(c\right)\;=\;\left\{\begin{array}{cc} \;0\;, & \;c=0\\ \;1\;, & \;c>0 \end{array}\right.\;$$

which counts the number of non-zero weights in *C* and will be relevant later in our analysis. Consider again two objects with C = (c,2−c). When *c* approaches zero, thus marginalizing the first object to an arbitrary degree, the effective number should approach one. However, this does not materialize in $N_{+}$ due to its discontinuity. In general, we require that the ENF cannot jump upon an arbitrarily small change of weights, namely:

(C) *Continuity:* $\;N=N\left[C\right]\;\;\mathrm{is}\;\mathrm{continuous}\;\mathrm{on}\;\;C_{N}\;,\;\forall \;N\;$

The properties discussed above define the set N of all effective number functions. However, there are dependencies among these requirements. In particular, it can be easily checked that the boundary condition (B1) is a consequence of (B2) and additivity. Similarly, (B) follows from (B1), (B2), symmetry and monotonicity. This leaves us with:

**Definition** **1.** *A real-valued function N = N[C] on C is called an effective number function (belongs to set N) if it is simultaneously additive* (A)*, symmetric* (S)*, continuous* (C)*, monotonic* (M${}^{-}$)*and satisfies the boundary condition* (B2).

**Figure 1:**
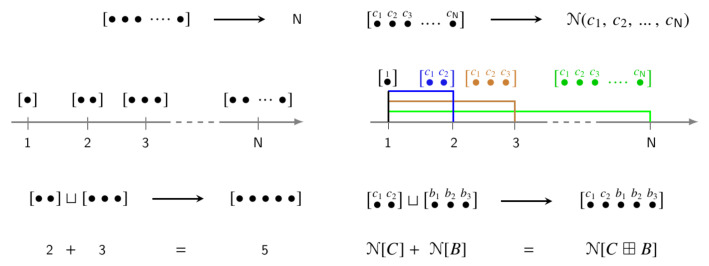
Schematic representation of the extension from natural numbers (**left**) to effective numbers (**right**). See the discussion in the text.

Some of the features imprinted on the corresponding notion of effective numbers are visualized in Figure 1 (remark [18]). On the left, natural numbers are shown as a theoretical model for expressing and manipulating the quantities of like objects (bags of balls) or of varied objects treated as equivalent. The bags containing differing amounts are assigned different discrete points on the real axis (natural numbers), with the operation of “merging the bags” (⊔) realized by ordinary addition. Extension to objects distinguished by counting weights is shown on the right. Here, the bags assigned equal amounts *N* by ordinary counting may be assigned different effective numbers N, depending on the cumulation of their weight distributions. With maximal cumulation (δ-function) producing N = 1, the effective number continuously and monotonically increases as cumulation decreases, reaching N = N when cumulation is absent (uniform distribution). The operation of merging bags is represented by the additivity property (A). Each element of N, if any, implements a specific version of this scheme. Thus, to assess the conceptual value and practical impact of effective numbers, it is necessary to decipher the structure of N.

### 1.2. Effective Counting

It is not difficult to establish that ENFs do exist. For example, one can verify that the one parameter family of functions:

(6) $$N_{\left(\alpha \right)}\left[C\right]=\sum_{i=1}^{N}n_{\left(\alpha \right)}\left(c_{i}\right)\;,\;n_{\left(\alpha \right)}\left(c\right)=\min\;\left\{c^{\alpha },1\right\}\;,\;0<\alpha \leq 1\;$$

belongs to N, with $N_{\left(1\right)}\;=\;N_{⋆}$. However, it is rather remarkable that all N∈N have the additively separable structure of (6). Indeed, Theorem 3 (Section 2) implies the following central result specifying N explicitly.

**Theorem** **1.** *Function N on C belongs to N if and only if there exists a real-valued function n=n(c) on [0,∞) that is concave, continuous, n(0)=0, n(c)=1 for c≥1, and:*

(7) $$N\left[C\right]\;=\;\sum_{i=1}^{N}n\left(c_{i}\right)\;,\;\forall \;C\in C_{N}\;,\;\forall \;N$$

*Such a function n associated with N∈N is unique.*

Thus, there is a one-to-one correspondence between ENFs and functions of the single variable specified by Theorem 1 (remark [19]). Such n associated with the given N will be referred to as its counting function.

The necessity of the additively separable form (7) for ENFs is interesting conceptually. Indeed, it is common and familiar to represent the ordinary total (natural number) by a sequential process of adding a unit amount for each object in the collection. According to Theorem 1, this applies to every consistent extension to the effective total (effective number), albeit with objects contributing weight-dependent amounts specified by the counting function. It thus turns out that the construction of ENFs generalizes the process of ordinary counting to the process of effective counting.

### 1.3. Minimal Effective Number

A key insight into the nature of effective counting is provided by the following results concerning the structure of set N. They follow directly from Theorem 4 in Section 2.

**Theorem** **2.** *Let $N_{⋆}\;\in N$ and $N_{+}\;\notin \;N$ be functions on C defined by* (1) *and* (5), *respectively. Then:*

$$\begin{array}{cc} \left(a\right)\;\; & \;N_{⋆}\left[C\right]\;\leq \;N\left[C\right]\;\leq \;N_{+}\left[C\right]\;\;,\;\;\forall \;N\in N\;\;,\;\;\forall \;C\in C\\ \left(b\right)\;\; & \;\left\{\;N[C]\;|\;N\in N\;\right\}\;=\;\left[\;\alpha ,\beta \right]\;\;,\;\;\alpha =N_{⋆}\left[C\right]\;\;,\;\;\beta =N_{+}\left[C\right]\;\;,\;\;\forall \;C\in C \end{array}$$

To elaborate, first note that (a) is the refinement of defining condition (B). While the upper bound is intuitive ($N_{+}\left[C\right]$ counts the number of non-zero weights in C), the lower one is unexpected and consequential. In particular, the effective number of objects weighted by *C* cannot be smaller than $N_{⋆}\left[C\right]$. Since $N_{⋆}$ is an ENF, this feature is inherent to the concept itself: there is a meaningful notion of the minimal effective number. In technical terms, $N_{⋆}$ is the least element of function set N with respect to partial order $\left(N_{1}\leq N_{2}\right)\;\Leftrightarrow \;\left(N_{1}\left[C\right]\leq N_{2}\left[C\right],\;\forall \;C\in C\right)$, and thus a unique ENF with this property.

Part (b) conveys that, for each fixed C ∈ C, effective counting can be adjusted so that N[C] assumes any desired value from the allowed range specified by (a). While reflecting a certain degree of arbitrariness built into the concept of effective numbers, the associated freedom of choice is in fact quite natural. To illustrate this, consider *N* objects with non-zero weights of very disparate magnitudes so that the collection is usefully characterized by an effective number. The insistence on the ordinary count in this situation constitutes a “large extrapolation” since it forces each object to contribute equally despite the disparity. According to (b), such an extrapolation can be realized by a sequence of ENFs that bring the effective total arbitrarily close to *N*. Accommodating the needed continuum of consistent schemes can thus be considered a useful feature in a framework describing the generalized aspects of counting.

Note that (b) also confirms an intuitive expectation that there is no maximal ENF since, although specifying a supremal value for each *C*, function $N_{+}$ does not belong to N. Taken together, the results of Theorem 2 form the basis for our canonical solution [A] of the quantum identity problem [Q]. The existence of minimal total $N_{⋆}$ is particularly consequential in applications of effective numbers. One notable example is that it facilitates the notion of minimal (intrinsic) quantum uncertainty [20].

## 2. Effective Number Theory

In this section, we will develop the theory of effective numbers with the requisite mathematical detail. The aim is to do this in a self-contained accessible manner using elementary mathematics. Certain generalizations regarding the underlying algebraic structure will be elaborated upon in a separate mathematical account.

### 2.1. Effective Complementary Numbers

It turns out that there are several practical advantages to carrying out this discussion in terms of effective complementary numbers (effective co-numbers) realized by functions (remark [21]):

(8) $$M\left[C\right]=N-N\left[C\right]\;,\;C\in C_{N}\;,\;N\in N$$

where N=N[C] are the ENFs introduced in Section 1.1. Following this route, we start by the explicit definition of effective co-number functions (co-ENFs) entailed by the above relationship.

**Definition** **2.** M *is the set of effective co-number functions M, where M: C→R have the following properties: for all $N,M\in Z^{+}$, for all integer 1≤i,j≤N, i≠j, for all $C=\left(c_{1},\ldots ,c_{N}\right)\in C_{N}$, and for all $B\in C_{M}$,*

(**A**) *additivity: M[C⊞B]=M[C]+M[B]*

(**co-B2**) *boundary values: M(N,0,…,0)=N−1, where $\left(N,0,\ldots ,0\right)\in C_{N}$*

(**C**) *continuity of M restricted to $C_{N}$ whose topology is inherited from the standard topology on $R^{N}$*

(**M${}^{+}$**) *monotonicity: $0<\varepsilon \leq \min\{c_{i},N-c_{j}\}$, $c_{i}\leq c_{j}\;\Rightarrow \;$$M\left(\ldots ,c_{i},\ldots ,c_{j},\ldots \right)\leq M\left(\ldots ,c_{i}-\varepsilon ,\ldots ,c_{j}+\varepsilon ,\ldots \right)$*

(**S**) *symmetry: $M\left(\ldots ,c_{i},\ldots ,c_{j},\ldots \right)=M\left(\ldots ,c_{j},\ldots ,c_{i},\ldots \right)$*

The following examples will be useful in the course of our analysis.

**Example** **1.** *The function $M_{\left(\alpha \right)}\left[C\right]=\sum_{i}m_{\left(\alpha \right)}\left(c_{i}\right)=\sum_{c_{i}=0}1+\sum_{c_{i}\in \left(0,1\right)}\left(1-c_{i}^{\alpha }\right)$, where:*

$$\begin{array}{c} m_{\left(\alpha \right)}\left(c\right)=\left\{\begin{array}{cc} 1, & c=0\\ 1-c^{\alpha }, & 0<c<1\\ 0, & 1\leq c \end{array}\right. \end{array}$$

*belongs to M for α∈(0,1]. This example is complementary to $N_{\left(\alpha \right)}$ introduced in (6).*

**Example** **2.** *The function $M_{+}\left[C\right]=M_{\left(0\right)}\left[C\right]=\sum_{i=1}^{N}m_{+}\left(c_{i}\right)$, where:*

$$\begin{array}{c} m_{+}\left(c\right)=m_{\left(0\right)}\left(c\right)=\left\{\begin{array}{cc} 1, & c=0\\ 0, & 0<c \end{array}\right. \end{array}$$

*satisfies Definition 2, except for continuity. Thus, $M_{+}\notin M$ is complementary to $N_{+}$ in (5).*

**Example** **3.** *The function $M_{⋆}=M_{\left(1\right)}\left[C\right]=\sum_{i=1}^{N}m_{⋆}\left(c_{i}\right)$, where:*

$$\begin{array}{c} m_{⋆}\left(c\right)=m_{\left(1\right)}\left(c\right)=\left\{\begin{array}{cc} 1-c, & 0\leq c<1\\ 0, & 1\leq c \end{array}\right. \end{array}$$

*satisfies all the properties from Definition 2, so $M_{⋆}\in M$. This co-ENF is complementary to $N_{⋆}$ in (1).*

Due to its repeated use in what follows, it is useful to formalize the following obvious Lemma.

**Lemma** **1.** *If M satisfies (A) and (S), then for all N,*

(**i**) *$M\left[C\right]=M\left(c_{1},\ldots ,c_{i-1},1,c_{i+1},\ldots ,c_{N}\right)=M\left(c_{1},\ldots ,c_{i-1},c_{i+1},\ldots ,c_{N}\right)+M\left(1\right)$, where $C\in C_{N}$,*

(**ii**) *M(1,…,1)=NM(1), where $\left(1,\ldots ,1\right)\in C_{N}$.*

### 2.2. Separability

We now focus on demonstrating the results that will ultimately clarify the content of set M and thus of set N. The main conclusion of the analysis here is that all co-ENFs are of an additively separable form, such as the one exhibited by the family of functions $M_{\left(\alpha \right)}$. The property of additive separability is defined as follows (remark [22]):

**Definition** **3.** *Additively separable function G on C is one that can be expressed as:*

(9) $$\begin{array}{c} G\left[C\right]=\sum_{i=1}^{N}g\left(c_{i}\right)\;,\;C\in C_{N}\;,\;N=1,2,\ldots \end{array}$$

*where g(c) is some function defined on [0,∞). Function g(c) is called a generating function of G[C].*

Additively separable G is generated by infinitely many distinct functions. However, for co-ENFs, a canonical representative can be singled out that is continuous and bounded on [0,∞) (see Proposition 2 and Corollary 2).

The relevant insight into additive separability is provided by Lemma 2 below. Before formulating it, let us associate with every $C\;\in \;C_{N}$ the vector $C^{↑}\;\in \;C_{N}$ obtained by permuting the components of *C* into ascending order. Furthermore, $C_{<}^{↑}$ will denote a vector obtained from $C^{↑}$ by keeping only components less than one and removing the rest. Note that for any symmetric function M on C, we have $M\left[C\right]=M\left[C^{↑}\right]$ and also that $C_{<}^{↑}\notin C$. In Proposition 1, we will work with $C_{\leq }^{↑}$ with an analogous meaning.

**Lemma** **2.** **(Separability)** *Let G be a function on C satisfying (A), (S), and the property:*

(10) $$\begin{array}{c} \forall \;N\;\;,\;\;\forall \;C\;,B\in C_{N}\;:\;C_{<}^{↑}\;=\;B_{<}^{↑}\;⟹\;G\left[C\right]=G\left[B\right] \end{array}$$

*Then, the following statements hold:*

(**a**) *G is additively separable.*

(**b**) *If, in addition, G is continuous (remark* [23]) *on $C_{2}$, then there exists a continuous function generating it.*

**Proof.**  (**a**) Assuming C≠(1,1,…,1), let $C^{↑}=\left(c_{1},\ldots ,c_{N}\right)\in C_{N}$ and $C_{<}^{↑}=\left(c_{1},\ldots ,c_{m}\right)$. We will distinguish two cases, namely 2m≤N and 2m>N, for which we respectively get by using (10):

$$\begin{array}{c} G\left[C\right]=G\left(c_{1},\ldots ,c_{m},c_{m+1},\ldots ,c_{N}\right)=G\left(c_{1},\ldots ,c_{m},2-c_{1},\ldots ,2-c_{m},1,\ldots ,1\right),\\ G\left[C⊞\left(1,\ldots ,1\right)\right]=G\left(c_{1},\ldots ,c_{m},c_{m+1},\ldots ,c_{N},1,\ldots ,1\right)=G\left(c_{1},\ldots ,c_{m},2-c_{1},\ldots ,2-c_{m}\right), \end{array}$$

with $2-c_{\ell }>1$ for ℓ=1,…,m. The vectors on the top line (case 2m≤N) are from $C_{N}$, while those on the bottom line (case 2m>N) are from $C_{2m}$. In both cases, Lemma 1, symmetry (S), and additivity (A) lead to:

G[C]=G(c1,2−c1,…,cm,2−cm) + (N−2m) G(1)=G(c1,2−c1)⊞…⊞(cm,2−cm) + (N−2m) G(1)=∑ℓ=1m FfG(cℓ,2−cℓ)−G(1)  + (N−m) G(1).

Consequently, introducing the generating function:

(11) $$\begin{array}{c} g\left(x\right)=\left\{\begin{array}{cc} G(x,2-x)-G(1), & x\in [0,1]\\ G(1), & x\in (1,\infty ) \end{array}\right. \end{array}$$

facilitates the claimed separability $G\left[C\right]=\sum_{i=1}^{N}g\left(c_{i}\right)$. Note that for C=(1,1,…,1), which was initially excluded, the separability holds in the same form.

(**b**) Given the proof of (a), it is sufficient to show that the continuity of G on $C_{2}$ implies the continuity of g in (11). For that, one only needs to ascertain the continuity at the gluing point x=1, which holds since we have two continuous functions with the same value at the gluing point: G(1,2−1)−G(1)=G(1). □

We will now demonstrate that all co-ENFs satisfy (10), and hence, they are additively separable.

**Proposition** **1.** *All functions M∈M are additively separable.*

**Proof.**  Because of symmetry (S), we will without loss of generality assume $C=C^{↑}$, i.e., *C* is in ascending order. For C=(1,1,…,1), the implication in (10) is vacuously true (remark [24]), so C≠(1,1,…,1) is assumed in what follows. We will use index *ℓ* to label the elements of $C_{\leq }^{↑}$ and index *j* for the rest of the entries in *C*. Hence, $c_{\ell }\leq 1<c_{j}$ for ℓ=1,…,m,  j=m+1,…,m+n=N, and $n=\sum_{j}1$. Then, by monotonicity (M${}^{+}$), Lemma 1, and M(1) = 0, which is the N = 1 case of (co-B2), we have:

M(…cℓ…,…cj…)≤M(…cℓ…,cm+1−ε,cm+2,…,cm+n−1,cm+n+ε), whereε=cm+1−1=M(…cℓ…,1,xxxxxicm+2,…,cm+n−1,1+(cm+n−1)+(cm+1−1))≤M(…cℓ…,1,xxcm+2−ε,cm+3,…,cm+n−1,1+(cm+n−1)+(cm+1−1)+ε)≤M…cℓ…,1,…,1,1+∑(cj−1)=M…cℓ…,xxxxxii1−n+∑cj.

The opposite inequality follows from additivity (A), (co-B2), and (M${}^{+}$). With ⌈x⌉ denoting the ceiling function, we start with $\sum (\left\lceil c_{j}\right\rceil -1)$ zeroes:

$$\begin{array}{c} M\left(\ldots c_{\ell }\ldots ,\;\ldots c_{j}\ldots \right)=M\left(\ldots c_{\ell }\ldots ,\;\ldots c_{j}\ldots ,\;0,\ldots ,0,\;1+\sum_{j=m+1}^{m+n}\left(\left\lceil c_{j}\right\rceil -1\right)\right)-\sum_{j=m+1}^{m+n}\left(\left\lceil c_{j}\right\rceil -1\right)\\ \geq M\left(\ldots c_{\ell }\ldots ,\;c_{m+1}+\varepsilon ,c_{m+2},\ldots ,c_{m+n},\;0,\ldots ,0,\;1+\sum \left(\left\lceil c_{j}\right\rceil -1\right)-\varepsilon \right)-\sum \left(\left\lceil c_{j}\right\rceil -1\right)\\ \geq M\left(\ldots c_{\ell }\ldots ,\;\left\lceil c_{m+1}\right\rceil ,\ldots ,\left\lceil c_{m+n}\right\rceil ,\;0,\ldots ,0,\;1+\sum \left(\left\lceil c_{j}\right\rceil -1\right)-\sum \left(\left\lceil c_{j}\right\rceil -c_{j}\right)\right)-\sum \left(\left\lceil c_{j}\right\rceil -1\right)\\ =M\left(\ldots c_{\ell }\ldots ,\;1+\;\sum_{j}\left(-1\right)-\sum \left(-c_{j}\right)\right)+\sum M\left(0,\ldots ,0,\;\left\lceil c_{j}\right\rceil \right)-\sum \left(\left\lceil c_{j}\right\rceil -1\right)\\ =M(\ldots c_{\ell }\ldots ,\;1-n+\sum c_{j}). \end{array}$$

The resulting equality implies (remark [25]) the property (10) upon recalling that the value of M does not change when removing the entries $c_{\ell }\;=\;1$ from *C*, so that $C_{\leq }^{↑}$ becomes $C_{<}^{↑}$. Moreover, since (A) and (S) are among the defining properties of co-ENFs, M is additively separable by Lemma 2 (a). □

**Corollary** **1.** *All functions M∈M satisfy* (10).

Next, we investigate the non-uniqueness of the generating function, which requires some groundwork to begin with.

**Lemma** **3.** *In real numbers, the following two statements are true:*

$$\begin{array}{cc} \left(i\right)\; & \left(\forall a)(\forall b>a)(\forall x\neq 1)\;(\exists m,n\in Z)(\exists z\in [a,b])\;(mx+nz=m+n\right)\\ \left(\mathrm{ii}\right)\; & \left(\forall a>1\right)\left(\forall b>a\right)\;\;\left(\exists B\right)\;\;\left(\forall x\in \left[0,\frac{1}{2}\right]\right)\;\;\left(\exists m,n\in Z^{+}\right)\left(\exists z\in \left[a,b\right]\right)\;\;\left(mx+nz=m+n,\;\;and\;\;\frac{n}{m}\leq B\right) \end{array}$$

**Proof.**  (**i**) Fit $\frac{m}{n}$ between $\frac{a-1}{1-x}$ and $\frac{b-1}{1-x}$ using the density of rationals in R. Then, to get the equality, choose $z=1+\left(1-x\right)\frac{m}{n}$.

(**ii**) Choose $B=\frac{1}{a-1}$ and $n=\lceil \frac{1}{b-a}\rceil$, then:

$$\frac{1}{n}\leq b-a\leq \frac{b-a}{1-x}=\frac{b-1}{1-x}-\frac{a-1}{1-x}$$

since 0≤x<1. Hence, there exists *m* to fit $\frac{m}{n}$ between $\frac{a-1}{1-x}$ and $\frac{b-1}{1-x}$. Then, again, choose $z=1+\left(1-x\right)\frac{m}{n}$ to get the equality. Moreover,

$$\left(a-1\right)\leq \frac{a-1}{1-x}\leq \frac{m}{n}\;\mathrm{and}\;\mathrm{so}\;\frac{n}{m}\leq \frac{1}{a-1}=B.$$

This concludes the proof. □

**Lemma** **4.** *Let G be an additively separable function on C and $g,g_{1},g_{2}$ its generating functions. Then,*

(**i**) *g(c)+(1−c)K is also a generating function of G for every number K,*

(**ii**) *if $g_{1}\left(c\right)-g_{2}\left(c\right)$ is bounded on some interval [a,b],0≤a<b, then $g_{1}\left(c\right)-g_{2}\left(c\right)=\left(1-c\right)K_{0}$ for some number $K_{0}\;$ and all c∈[0,∞).*

**Proof.**  (**i**) This follows from $\sum_{i=1}^{N}\left(1-c_{i}\right)=0$ for all $C=\left(\ldots c_{i}\ldots \right)\in C_{N}$.

(**ii**) Setting $\tilde{g}=g_{1}-g_{2}$ gives the following equation:

(12) $$\sum \tilde{g}\left(c_{i}\right)=0\;\mathrm{for}\;\mathrm{all}\;N\;\mathrm{and}\;\mathrm{all}\;C=\left(c_{1},\ldots ,c_{N}\right).$$

We will show that:

(13) $$\tilde{g}\left(c\right)=\left(1-c\right)\;\tilde{g}\left(0\right)\;\mathrm{for}\;\mathrm{all}\;c\in \left[0,\infty \right).$$

Case c=1. We get $\tilde{g}\left(1\right)=0$ from (12) for N=1, and then, (13) is satisfied for c=1.

Case c>1. Without loss of generality, we can assume that 1∉[a,b]. Moreover, if 0≤a<b<1, then $\tilde{g}$ is bounded also on [2−b, 2−a] because $\tilde{g}\left(2-c\right)=-\tilde{g}\left(c\right)$, which follows from (12) when N=2. As a result, we can assume without loss of generality even that 1<a<b. Under this assumption, we will first show that $\tilde{g}$, bounded on [a,b], must be bounded also on $\left[0,\frac{1}{2}\right]$. According to Lemma 3(ii), we have:

$$\left(\forall a>1\right)\left(\forall b>a\right)\;\left(\exists B\right)\;\left(\forall x\in \left[0,\frac{1}{2}\right]\right)\;\left(\exists m,n\in Z^{+}\right)\left(\exists z\in \left[a,b\right]\right)\;\left(mx+nz=m+n,\;\;\mathrm{and}\;\;\frac{n}{m}\leq B\right)$$

Now, we choose $C=\left(x,\ldots ,x,z,\ldots ,z\right)\in C_{m+n}$ in Equation (12), where *x* repeats *m* times and *z* repeats *n* times. Then:

$$m\tilde{g}\left(x\right)+n\tilde{g}\left(z\right)=0\;\mathrm{and}\;\mathrm{so}\;\left|\tilde{g}\left(x\right)\right|=\left|\frac{-n}{m}\tilde{g}\left(z\right)\right|\leq BA\;,\;\mathrm{where}$$

*A* is a bound for $|\tilde{g}|$ on [a,b]. Thus, we have $\tilde{g}$ bounded on $\left[0,\frac{1}{2}\right]$ by BA.

Now, suppose by contradiction that there is c>1 such that $\tilde{g}\left(c\right)\neq \left(1-c\right)\;\tilde{g}\left(0\right)$. Let *k* be an integer large enough that:

(14) $$N-k-2=\left\lceil kc\right\rceil -k-2\geq 0\;\mathrm{and}\;\frac{4BA}{k}<\left|\tilde{g}\left(c\right)-\left(1-c\right)\tilde{g}\left(0\right)\right|$$

and set N=⌈kc⌉, $x=\frac{1}{2}\left(N-kc\right)$. Then, kc+2x=N and $x\in [0,\frac{1}{2}]$. Choosing $C=\left(c,\ldots ,c,0,\ldots ,0,x,x\right)\in C_{N}$ in (12), where *c* repeats *k* times, will produce:

$$k\tilde{g}\left(c\right)+\left(N-k-2\right)\;\tilde{g}\left(0\right)+2\tilde{g}\left(x\right)=0$$

and the first condition in (14) ensures that *C* will not have a negative number of zeroes. Given that N=kc+2x, we get:

$$\begin{array}{c} \tilde{g}\left(c\right)+\left(c-1+\frac{2x}{k}-\frac{2}{k}\right)\;\tilde{g}\left(0\right)+\frac{2\tilde{g}\left(x\right)}{k}=0 \end{array}$$

(15) $$\begin{array}{c} \tilde{g}\left(c\right)=\left(1-c\right)\;\tilde{g}\left(0\right)+\varepsilon , \end{array}$$

where we set $\varepsilon =-\frac{2}{k}\left(\left(x-1\right)\;\tilde{g}\left(0\right)+\tilde{g}\left(x\right)\right)$. Since kε is bounded by 4BA and from the second condition in (14), we have:

$$\begin{array}{c} 0<|\varepsilon |\leq \frac{4BA}{k}<\left|\tilde{g}\left(c\right)-\left(1-c\right)\;\tilde{g}\left(0\right)\right|, \end{array}$$

which is a contradiction with (15). Thus, the original assumption $\tilde{g}\left(c\right)\neq \left(1-c\right)\;\tilde{g}\left(0\right)$ failed, and (13) holds for c>1.

Case c<1. We can use the previous case for 2−c>1 and get $\tilde{g}\left(2-c\right)=\left(1-\left(2-c\right)\right)\;\tilde{g}\left(0\right)$. This equation transforms into (13) since we already know that $\tilde{g}\left(2-c\right)=-\tilde{g}\left(c\right)$, and thus, (13) holds for c<1 as well.

We have shown that if $\tilde{g}$ satisfies (12), then it satisfies (13). Finally, setting $K_{0}=\tilde{g}\left(0\right)$ completes the proof. □

**Proposition** **2.** *Let M∈M. Then, for each number t, there is a unique generating function m of M that is continuous, and m(0)=t.*

**Proof.**  The existence of one continuous generating function, not necessarily satisfying m(0)=t, follows from Corollary 1 and Lemma 2(b). Then part (i) of Lemma 4 implies that there is at least one continuous generating function for an arbitrary value of t=m(0). To show the uniqueness of such a function for every *t*, assume that there are two continuous generating functions $m_{1}$ and $m_{2}$, such that $m_{1}\left(0\right)=m_{2}\left(0\right)$. Since $m_{1}\left(c\right)-m_{2}\left(c\right)$ is then bounded on any finite interval due to continuity, we can use (ii) of Lemma 4 to infer that $0=m_{1}\left(0\right)-m_{2}\left(0\right)=K_{0}$. Using (ii) of Lemma 4 again, we finally conclude $m_{1}\left(c\right)=m_{2}\left(c\right)$, as claimed. □

### 2.3. Description and Structure of Co-ENFs

The separability results of the previous section give us access to the content and the structure of set M, ultimately providing a key insight into the concept of effective (co-)numbers. We start with the following proposition (remark [26]):

**Proposition** **3.** (**i**) *Let G be a real additively separable function defined on C. G is continuous (C) and monotone (M${}^{+}$) if and only if it can be generated by a function g(c) that is continuous at c=0 and convex.*

(**ii**) *If M∈M, then all its continuous generating functions m(c) are convex.*

**Proof.**  (**i**) (**⇐**) The convexity and continuity of g at c=0 imply its continuity on [0,∞), which guarantees the continuity (C) of G. In the presence of additive separability, conditions entailed by (M${}^{+}$) take the form:

(16) $$\begin{array}{c} g\left(c_{i}\right)+g\left(c_{j}\right)\leq g\left(c_{i}-\varepsilon \right)+g\left(c_{j}+\varepsilon \right)\;,\;c_{i}\leq c_{j} \end{array}$$

To show that this also follows from the stated properties of g, consider function $\tilde{g}$, which equals g everywhere except on interval $\left[c_{i},c_{j}\right]$, where it is replaced by a linear segment with boundary values $g(c_{i})$ and $g(c_{j})$. Such a function $\tilde{g}$ is still convex, which implies:

$$\begin{array}{c} \tilde{g}\left(\frac{\left(c_{i}-\varepsilon \right)+\left(c_{j}+\varepsilon \right)}{2}\right)\leq \frac{\tilde{g}\left(c_{i}-\varepsilon \right)+\tilde{g}\left(c_{j}+\varepsilon \right)}{2}. \end{array}$$

Then, by linearity, the left-hand side is:

$$\tilde{g}\left(\frac{c_{i}+c_{j}}{2}\right)=\frac{\tilde{g}\left(c_{i}\right)+\tilde{g}\left(c_{j}\right)}{2}=\frac{g\left(c_{i}\right)+g\left(c_{j}\right)}{2}.$$

The inequality turns into:

$$\begin{array}{c} g\left(c_{i}\right)+g\left(c_{j}\right)\leq \tilde{g}\left(c_{i}-\varepsilon \right)+\tilde{g}\left(c_{j}+\varepsilon \right)=g\left(c_{i}-\varepsilon \right)+g\left(c_{j}+\varepsilon \right) \end{array}$$

as needed.

(**⇒**) Consider the (M${}^{+}$) condition $G\left(\ldots c_{i}\ldots c_{j}\ldots \right)\leq G\left(\ldots c_{i}-\varepsilon \ldots c_{j}+\varepsilon \ldots \right)$ for additively separable G. Setting $c_{i}=c_{j}=c$ and, subsequently, a=c−ε,b=c+ε, we obtain in turn:

$$\begin{array}{ccc} g(c)+g(c) & \leq & g(c-\varepsilon )+g(c+\varepsilon )\\ g\left(\frac{a+b}{2}\right) & \leq & \frac{g(a)+g(b)}{2}. \end{array}$$

Hence, any g, a generating function of G, is midpoint convex on [0,N] for all *N*. It is well known that every such function is convex if it is continuous. We thus select a continuous generating function g, whose existence is guaranteed by Proposition 12. Such a resulting g is then both continuous at c=0 and convex. Note that g is an arbitrary continuous generating function, so all continuous generating functions g are convex. This is needed in the proof of (ii) that follows.

(**ii**) Proposition 1 implies the additive separability of M∈M, and the rest of the demonstration is contained in the proof of (i)(⇒) above. □

We are now in a position to describe the set M, specified by Definition 2, explicitly.

**Theorem** **3.** **(Set of co-ENFs)**

*M∈M if and only if it is generated by a convex and continuous function m, which is zero on [1,∞) and m(0)=1. Such a generating function m of M is unique.*

**Proof.**  (**⇒**) M∈M is additively separable by Proposition 1. Then, as a consequence of additivity (A) and the boundary conditions (co-B2), we have M(0,…,0,N)=(N−1)·m(0)+m(N)=N−1, so that:

(17) $$\begin{array}{c} m\left(N\right)=\left(N-1\right)\cdot \left(1-m(0)\right) \end{array}$$

for all *N*. Given the continuity of M∈M, Proposition 2 guarantees the existence of its unique continuous generating function with m(0)=1. In conjunction with Equation (17), this implies that m(N)=0 for all *N*. Furthermore, this continuous generating function is convex by (ii) of Proposition 3, and consequently, it is zero on the entire [1,∞). This demonstrates the existence of unique m with all required properties.

(**⇐**) For the opposite direction, let $M\left[C\right]=\sum m\left(c_{i}\right)$, where m is continuous, convex, m(0)=1, and m(c)=0 on [1,∞). Then (A), (C), (S), and (co-B2) follow immediately, while (M${}^{+}$) is a consequence of Proposition 3(i). □

Note that the unique choice of the continuous generating function for co-ENF is facilitated by a natural choice m(0)=1, expressing the fact that the object assigned zero probability should not contribute to the effective number total (n(0)=0). However, it is worth pointing out that, as shown below, the same unique choice of a generating function is selected by the requirement of the boundedness on the entire [0,∞).

**Corollary** **2.** *Let m be the generating function of M∈M, specified in Theorem 3. Then:*

(**i**) *0≤m(c)≤1 for all c*

(**ii**) *m is the only generating function of M that is bounded on its whole domain [0,∞).*

**Proof.**  (**i**) This immediately follows from m(0)=1, m(c)=0 on [1,∞), and convexity.

(**ii**) The boundedness of m follows from (i). To demonstrate uniqueness, assume there is another bounded generating function $m_{1}$ of M. Thus, $m_{1}-m$ satisfies the assumptions of Lemma 4(ii), implying the existence of non-zero $K_{0}$ such that $m_{1}\left(c\right)=m\left(c\right)+K_{0}\left(1-c\right)$ for c∈[0,∞). However, this contradicts the boundedness of $m_{1}$, which demonstrates the claimed uniqueness. □

Below, we will make use of the following obvious lemma and a simple corollary.

**Lemma** **5.** *Let $G_{1}$ and $G_{2}$ be real additively separable functions on C. If there exist respective generating functions such that $g_{1}\left(c\right)\leq g_{2}\left(c\right)$, for all c, then $G_{1}\left(C\right)\leq G_{2}\left(C\right)$, for all C∈C.*

**Corollary** **3.** *If M∈M, then 0≤M[C]≤N−1, for all C∈C.*

**Proof.**  Let m be the generating function specified in Theorem 3. From (i) of Corollary 2, we have $\sum_{i}0\leq \sum m\left(c_{i}\right)\leq \sum_{i}1$, which translates into 0≤M[C]≤N by Lemma 5. To put the second inequality into the claimed form, note that there is always at least one $c_{j}\geq 1$. For this $c_{j}$, we have $m(c_{j})=0$ by Theorem 3. This lowers the upper bound for M[C] by unity and proves the second inequality. □

Using the above preparation, we will now demonstrate several structural properties of M.

**Theorem** **4.** **(Maximality)**

*If M∈M, then the following holds for all $C=(\ldots ,c_{i},\ldots )\in C$,*

(**i**) *$M_{\left(0\right)}\left[C\right]\;=\;M_{+}\left[C\right]\;\leq \;M\left[C\right]\;\leq \;M_{⋆}\left[C\right]\;=\;M_{\left(1\right)}\left[C\right]$*

(**ii**) *$M_{+}\left[C\right]\;=\;M\left[C\right]\;=\;M_{⋆}\left[C\right]\;\Leftrightarrow \;c_{i}\notin \left(0,1\right),\;i=1,2,\ldots ,N$*

(**iii**) *$\beta_{0}\;=\;M_{+}\left[C\right]\;<\;M_{⋆}\left[C\right]\;=\;\beta_{1}\;\;\Rightarrow \;\;\left\{M\left[C\right]:M\in M\right\}=\left[\beta_{0},\beta_{1}\right]$.*

**Proof.**  (**i**) To show both inequalities, let m be the continuous generating function of M guaranteed by Theorem 3. We will show that m satisfies $m_{+}\left(x\right)\leq m\left(x\right)\leq m_{⋆}\left(x\right)$ on [0,∞), and then, Lemma 5 will complete the proof of this part. The first inequality $m_{+}\left(x\right)\leq m\left(x\right)$ follows directly from Theorem 3, Corollary 2(i), and the definition of $m_{+}$. The second inequality holds as the equality on [1,∞) by Theorem 3 and the definition of $m_{⋆}$. To show the second inequality on [0,1), note that the graphs of both m and $m_{⋆}$ pass through the points (0,1) and (1,0), m is convex by Proposition 3(ii), and $m_{⋆}$ is linear between those points. Therefore, $m_{+}\left(x\right)\leq m\left(x\right)\leq m_{⋆}\left(x\right)$ on [0,∞), as promised.

(**ii**) (**⇐**) If $c_{i}\notin \left(0,1\right)$ for i=1,2,…,N, then $M_{+}\left[C\right]=M_{⋆}\left[C\right]$, and the equality for M[C] follows from (i).

(**⇒**) Let $M_{+}\left[C\right]=M_{⋆}\left[C\right]$. Then,

$$\sum_{c_{i}=0}1=\sum_{c_{i}=0}1+\sum_{c_{i}\in \left(0,1\right)}\left(1-c_{i}\right).$$

Hence, $\;\sum_{c_{i}\in \left(0,1\right)}\left(1-c_{i}\right)=0$, and so, $c_{i}\notin \left(0,1\right)$ for i=1,2,…,N.

(**iii**) From $M_{+}\left[C\right]<M_{⋆}\left[C\right]$, we have the existence of at least one $c_{i}\in \left(0,1\right)$ by (ii). Then, for a fixed *C*, define:

$$g\left(\alpha \right)=\sum_{c_{i}=0}1+\sum_{c_{i}\in \left(0,1\right)}\left(1-c_{i}^{\alpha }\right)=M_{\left(\alpha \right)}\left[C\right].$$

The function *g* is continuous and increasing and maps interval [0,1] onto interval $\left[\beta_{0},\beta_{1}\right]$. Then:

$$\left\{M\left[C\right]:M\in M\right\}⊇\left\{M_{\left(\alpha \right)}\left[C\right]:\alpha \in \left[0,1\right]\right\}=\left[M_{+}\left[C\right],\;M_{⋆}\left[C\right]\right]=\left[\beta_{0},\beta_{1}\right].$$

The opposite inclusion follows from (i). □

## 3. Counting the General Quantum Identities

With the effective number theory in place, we now return to the topic that motivated its construction, namely the quantum identity problem. In particular, we will make explicit some of the straightforward, but useful and relevant, generalizations of [Q]. This serves, in part, as a stepping stone toward the most generic application of effective numbers in quantum theory, namely as a tool to characterize quantum states (Section 4).

A conceptually important extension of [Q] and [A] is made possible by the additive separability of ENFs. Indeed, instead of an orthonormal basis, consider any collection {∣ j 〉} of *n* orthonormal states from an *N*-dimensional Hilbert space (1 ≤ n ≤ N). How many states from {∣ j 〉} is a system described by ∣ ψ 〉 effectively in? Let N∈N be an ENF and n the counting function uniquely associated with it by virtue of Theorem 1. We define:

(18) $$N\left[|\;\psi \;\rangle ,\{|\;j\;\rangle \}\;\right]\;\equiv \;\sum_{j=1}^{n}n\left(c_{j}\right)\;\;,\;\;c_{j}=N|\langle j|\psi \rangle {|}^{2}$$

for each ∣ ψ 〉 and {∣ j 〉}. This assignment is meaningful in the following sense. Given a fixed {∣ j 〉}, let {∣ i 〉} be its arbitrary completion into a basis of the Hilbert space. Then, owing to the additive separability of ENFs,

(19) $$N\left[|\;\psi \;\rangle ,\{|\;i\;\rangle \}\right]\;=\;N\left[\;|\;\psi \;\rangle ,\{|\;j\;\rangle \}\;\right]\;+\;N\left[\;|\;\psi \;\rangle ,\{|\;i\;\rangle \}\backslash \{|\;j\;\rangle \}\right]$$

where “\” denotes the set subtraction. In other words, the contribution of {∣ j 〉} to N[ ∣ ψ 〉,{∣ i 〉} ], defined by (18), is independent of the completion {∣ i 〉} and is thus uniquely associated with {∣ j 〉} for fixed N∈N. It is the effective number of states from {∣ j 〉} contained in ∣ ψ 〉 according to N. Moreover, it is straightforward to check that $N_{⋆}\left[\;|\;\psi \;\rangle ,\left\{\;|\;j\;\rangle \right\}\;\right]$ minimizes the effective number so assigned, and we have:

[A${}^{\prime }$] *Let ∣ ψ 〉 be a state vector from an N-dimensional Hilbert space and {∣ j 〉}≡{∣ j 〉∣ j=1,2,…,n≤N } the set of n orthonormal states in this space. The physical system described by ∣ ψ 〉 is effectively in $N_{⋆}\left[\;|\;\psi \;\rangle ,\left\{|\;j\;\rangle \right\}\right]$ states from {∣ j 〉}, specified by (18) with $n=n_{⋆}$.*

A few simple points regarding the above are worth emphasizing.

(i) The extension (18) and the ensuing generalization of [A] to [A${}^{\prime }$] arise because the abundance of quantum identities is determined “locally”, namely without reference to basis elements orthogonal to the subspace in question. Apart from being natural for a measure-like characteristic, this feature has practical consequences in many-body applications where the dimension of the Hilbert space grows exponentially with the size of the system. Indeed, the above avoids such complexity in certain calculations, thus providing a computational benefit.
(ii) None of the above applies to the abundance of quantum identities determined by the participation number $N_{p}$ of Equation (4), since this value does not split into contributions from orthogonal subspaces generated by the partitioned basis. This is of course due to the lack of additivity and hence of additive separability.
(iii) The above considerations are clearly not limited to counting quantum identities. In a generic situation, the inquiry is concerned with the contribution to the effective number from a subset of weighted objects. To formalize such assignments directly in the effective number theory, one simply extends N∈N from domain C of counting vectors to the domain of general weights:
  (20) $$W=\cup_{n}W_{n}\;,\;W_{n}\;\equiv \;\left\{\;W=\left(w_{1},w_{2},\ldots ,w_{n}\right)\;|\;w_{j}\geq 0\;\right\}\;$$
  by virtue of its counting function n, namely $N\left[W\right]=\sum_{j}n\left(w_{j}\right)$.

While the effective number N[ ∣ ψ 〉,{∣ i 〉}] specifies how many identities from basis {∣ i 〉} the state ∣ ψ 〉 can effectively take, it is frequently useful to inquire about a coarse-grained version of such an effective count. To formalize the corresponding generalization, consider the decomposition of Hilbert space H into *M* mutually orthogonal subspaces $\left\{H_{m}\right\}$:

(21) $$H=H_{1}⊕H_{2}⊕\ldots ⊕H_{M}\;,\;\left\{H_{m}\right\}\equiv \left\{H_{m}|\;m=1,2,\ldots ,M\;\right\}$$

Subspaces $H_{m}$ are implicitly treated as equivalent entities (remark [27]). In how many subspaces from $\left\{H_{m}\right\}$ is the system described by ∣ ψ 〉 effectively?

Given a state of the system, the rules of quantum mechanics assign a probability to each subspace of the associated Hilbert space. The effective number theory therefore provides an immediate answer to the above question. More specifically, let $|\;\chi_{m}\;\rangle$ be the (unnormalized) projection of ∣ ψ 〉 onto $H_{m}$. Then, the probability vector is $P=(p_{1},p_{2},\ldots ,p_{M})$, where $p_{m}=\langle \;\chi_{m}\;|\;\chi_{m}\;\rangle$, and the corresponding counting vector is C=MP. Hence, given an ENF, we assign $N\left[\;|\;\psi \;\rangle ,\left\{\;H_{m}\right\}\right]\equiv N\left[C\right]$, which leads to the following generalization [A${}_{g}$] of [A].

[A${}_{g}$] *Let C be the counting vector assigned by quantum mechanics to state ∣ ψ 〉 and the orthogonal decomposition $\left\{H_{m}\right\}$ of the Hilbert space. Then, the system described by ∣ ψ 〉 is effectively contained in $N_{⋆}\left[\;|\;\psi \;\rangle ,\left\{H_{m}\right\}\;\right]=N_{⋆}\left[C\right]$ subspaces from $\left\{H_{m}\right\}$.*

Applying the logic identical to the one producing [A${}^{\prime }$], it is straightforward to generalize [A${}_{g}$] into [A${}_{g}^{\prime }$] for counting the identities from arbitrary sets (not necessarily full decompositions) of mutually orthogonal subspaces.

## 4. Application: The Structure of Quantum States

While our discussion was carried out in the context of the quantum identity problem, the constructed effective number framework offers a much larger scope of uses. Apart from physics and mathematics, these also appear in the areas of applied science simply due to the very basic role of the measure and probability in quantitative analysis. Examples of such applications will be discussed in the follow-up works. Here, we describe a broader outlook on the utility of effective numbers in quantum theory.

With the quantum state encoding all “options” for the system, the physical content of ∣ ψ 〉 closely relates to all probability vectors *P* induced in this manner. Retracing the steps leading to [A], it is then meaningful to associate the effective number $N_{⋆}$ with any complete set of mutually exclusive possibilities. We propose the collection of all such reductions:

(22) $$|\;\psi \;\rangle \;⟶\;P\;⟶\;N_{⋆}$$

as a systematic and physically relevant characterization of a quantum state. The general quantum identities of Section 3, which can also be thought of as measurement outcomes, are the prime examples of objects/possibilities in question. However, any meaningful ∣ ψ 〉→P can be considered. The resulting variety offers a wide range of options for targeted insight into the structure of ∣ ψ 〉. We wish to highlight a few points in this regard.

(i) Each effective number characteristic can be refined by totals assigned to subsets of the associated sample space or coarse-grained by counting its partitions. Note that for quantum identities in [Q], this corresponds to refining by totals involving parts of the basis and coarse-graining by totals involving orthogonal decompositions.
(ii) The proposed approach is universal with respect to the nature of the quantum system being described. Indeed, ∣ ψ 〉 may be as simple as a state of the harmonic oscillator, but as complex as a many-body state of quantum spins or the vacuum of non-Abelian gauge theory.
(iii) Our present focus on the finite discrete case is not restrictive either. Even in situations involving the space-time continuum, the intermediate steps of ultraviolet (lattice) and infrared (volume) regularizations yield such descriptions. Removing the cutoffs then involves a suitable N → ∞ limit. For this purpose, it is more convenient to work with the effective fraction rather than the effective number. In terms of probabilities, it is defined as:
  (23) $$F\left[P\right]\;\equiv \;\frac{1}{N}\;N\left[NP\right]\;,\;N\in N\;,\;P\in P_{N}\;$$
  Function $N_{⋆}$ produces the minimal effective fraction, namely:
  (24) $$F_{⋆}\left[P\right]=\sum_{i=1}^{N}f_{⋆}\left(p_{i},N\right)\;,\;f_{⋆}\left(p,N\right)\equiv \min\;\left\{p,1/N\right\}$$
  Restricting ourselves to quantum identities of [Q], the cutoff removal schematically proceeds as follows. Assume that ∣ ψ 〉 is the target state (from the infinite-dimensional Hilbert space) to be assigned an effective fraction in basis {∣ i 〉}. Let $|\;\psi^{\left(k\right)}\;\rangle$ represent ∣ ψ 〉 at the *k*-th step of the regularization process, involving the state space of increasing dimension $N_{k}$. If the latter is spanned by $\left\{|\;i^{\left(k\right)}\;\rangle \right\}$ targeting {∣ i 〉}, then (remark [28]):
  (25) $$F_{⋆}\left[\;|\;\psi \;\rangle ,\;\{|\;i\;\rangle \}\;\right]\;\equiv \;\lim_{k\to \infty }F_{⋆}\left[\;|\;\psi^{\left(k\right)}\;\rangle ,\;\{|\;i^{\left(k\right)}\;\rangle \}\;\right]\;=\;\lim_{k\to \infty }F_{⋆}\left[P_{k}\right]$$
  where $P_{k}\;=\;\left(p_{1}^{\left(k\right)},\ldots ,p_{N_{k}}^{\left(k\right)}\right)$, $p_{i}^{\left(k\right)}\;=\;\;|\;\langle \;i^{\left(k\right)}\;|\;\psi^{\left(k\right)}\;\rangle \;{|}^{2}$. Note that $F_{⋆}$ reflects the “localization” of ∣ ψ 〉 in {∣ i 〉}.
(iv) An example of an application where the assignment (22) is carried out without direct reference to the underlying Hilbert space is provided by the problem of the vacuum structure in quantum chromodynamics (QCD). This is often studied in the Euclidean path integral formalism, with the regularized vacuum represented by the statistical ensemble of lattice gauge configurations $U\;=\;\{U_{x,\mu }\}$. Given a composite field O = O(x,U) and the induced space-time probability distribution P(x,U)∝|O(x,U)|, the effective number framework can be used to determine the effective fraction of space-time occupied by *O* for each *U*. The corresponding quantum averages are of vital interest in this context. Yet, more indirect vacuum characteristics of such a type reflect the space-time properties of Dirac eigenmodes, whose main utility is to probe the features of quark dynamics.

## 5. Concluding Remarks

When a quantum system is (strongly) probed, it emerges in one of many possible “identities”. This is among the key features of quantum behavior. Indeed, it underlies the notion of quantum uncertainty and is closely connected to a fruitful concept of localization. A well-founded prescription for the corresponding abundances is thus desirable. As a contemporary example, one may use it in the analysis of a quantum algorithm that produces the state ${|\;\psi \;\rangle }_{o}$ as an output of a quantum run and follows up with a measurement involving a basis {∣ i 〉}. The effective number of distinct collapsed states ∣ i 〉 obtained upon repetition of these steps is relevant for the assessment of the algorithm’s efficiency.

In this work, we showed that requiring the desired characteristics to be measure-like (additive) is fruitful in identifying a meaningful quantifier. In particular, it results in the theoretical structure (effective number theory) revealing that a consistent assignment of totals to collections of objects with probability weights requires the existence of an inherent (minimal) amount $N_{⋆}$. The appearance of such a qualitative feature in basic measure considerations suggests the utility of the constructed framework already in contexts much less abstract than quantum mechanics. For example, $N_{⋆}$ can be viewed as an extension of the ordinary counting measure into what can be referred to as a diversity measure with its wide range of contemporary applications (social sciences, ecosystems; see, e.g., [29,30]). Other viewpoints can cast it as a choice measure, facilitating a probabilistic notion of effective choices, or as a support measure, conveying the effective size of a function support (effective domain). Given this universality, $N_{⋆}$ may find uses in multiple areas of quantitative science.

Finally, we wish to point out that the existence of a minimal amount is rooted in the simultaneous requirement of both monotonicity (Schur concavity) and additivity for ENFs. This combination is rather unusual from the mathematics standpoint. Indeed, while monotonicity is important for the theory of majorization [31,32], it has no role in the standard formalization of the measure. Conversely, additivity is crucial for the latter, but not native to the former. In fact, relaxing either (M${}^{-}$) or (A) in Definition 1 leaves the respective effective pseudo-number assignments too arbitrary. However, their combination, which is necessary on conceptual grounds, leads to $N_{⋆}$ and the associated insights.

## References

[1] Anderson P.W. (1958). Absence of Diffusion in Certain Random Lattices. Phys. Rev..

[2] Markos P. (2006). Numerical Analysis of the Anderson Localization. Acta Phys. Slovaca.

[3] Evers F., Mirlin A.D. (2008). Anderson transitions. Rev. Mod. Phys..

[4] Heisenberg W. (1927). Über den anschaulichen Inhalt der quantentheoretischen Kinematik und Mechanik. Z. Phys..

[5] Kennard E.H. (1927). Zur Quantenmechanik einfacher Bewegungstypen. Z. Phys..

[B6-entropy-22-01273] 6.Note that this canonical setting already covers many-body and field-theoretic systems whose quantum dynamics can be defined via lattice regularization. The extension to continuously labeled bases will be explicitly given in the context of the application to quantum uncertainty (item 20 in this reference list).

[B7-entropy-22-01273] 7.This is rooted in the fact that quantifiers of the desired type cannot be expressed as quantum-mechanical expectation values in state ∣*ψ*〉.

[B8-entropy-22-01273] 8.ENFs would still be useful since, by construction, each of them individually conveys a universal comparative information about effective totals.

[B9-entropy-22-01273] 9.The existence of multiple ENFs endows the constructed framework with flexibility to accommodate quantum identity problems more structured than [Q]. This may entail an additional problem-specific constraint(s) on an ENF, possibly leading to a unique or privileged choice other than 𝒩_⋆_. However, a generic extra requirement is that the effective total determines the subset of {∣*i*〉} in which ∣*ψ*〉 is effectively present. For example, in the context of Anderson localization, it is of interest to identify the spatial region effectively occupied by the electron. It can be shown that 𝒩_⋆_ is the only ENF leading to a consistent selection of such effective support of ∣*ψ*〉 on {∣*i*〉}.

[B10-entropy-22-01273] 10.Working with counting vectors (2) rather than probability vectors *P* ∈ *𝒫* = ∪_*N*_ *𝒫_N_* is simply a matter of convenience. All results translate straightforwardly.

[B11-entropy-22-01273] 11.If *C* = (*c_1_*,…,*c_N_*) ∈ *𝒞_N_* and *B* = (*b_1_*,…,*b_M_*) ∈ *𝒞_M_*, then *C* ⊞ *B* ≡ (*c_1_*,…,*c_N_*,*b_1_*,…,*b_M_*).

[12] Bell R.J., Dean P. (1970). Atomic vibrations in vitreous silica. Disc. Faraday Soc..

[13] Shannon C.E. (1948). A Mathematical Theory of Communication. Bell Syst. Technol. J..

[14] Rényi A. On Measures of Entropy and Information. Proceedings of the 4th Berkeley Symposium on Mathematical Statistics and Probability.

[B15-entropy-22-01273] 15.We write 𝒩(c_1_,…,c*_N_*) when weights need to be distinguished, but use the functional notation 𝒩[C] otherwise.

[B16-entropy-22-01273] 16.To visualize how the elementary deformation in (M^−^) increases cumulation, one may picture each object as a cylindrical column of incompressible liquid in the amount of its counting weight. Arranging the columns by increasing height from the left to the right produces a half-peak profile with cumulation on the right. Consider the segment of this profile delimited by columns *c_i_* and *c_j_* entering (M^−^). The monotonicity operation is represented by a transverse flow of liquid from the left to the right endpoint through the columns between them. It is understood that the columns are ordered at every moment of the flow, and thus, as the amount of liquid at the endpoints changes, the length of the segment may increase. Since the liquid flows toward the center of cumulation at every point of the process, the resulting distribution is more cumulated than the original one.

[B17-entropy-22-01273] 17.Monotonicity (M^−^) is closely related to Schur concavity. The latter is equivalent to imposing (M^−^) and symmetry (S) simultaneously (see, e.g., item 31 in this reference list).

[B18-entropy-22-01273] 18.Once an ENF is fixed and used to assign totals, the conventional “number of objects” is replaced by the “effective number of objects”. While we use the term effective number only in this restricted sense here, the underlying algebraic structure makes the concept similar to standard types of numbers.

[B19-entropy-22-01273] 19.Note that it suffices to require continuity at *c* = 0 since concavity guarantees it elsewhere.

[20] Horváth I. (2018). The Measure Aspect of Quantum Uncertainty, of Entanglement, and the Respective Entropies. arXiv.

[B21-entropy-22-01273] 21.They could also be called effective dual numbers, but the duality is not a central property here.

[B22-entropy-22-01273] 22.Unless stated otherwise, referencing “function” in this section applies to both real and complex-valued functions, and referencing “number” applies to both real and complex options.

[B23-entropy-22-01273] 23.The continuity on *𝒞*_2_ may appear weaker than (*C*) continuity, but this lemma shows that they are equivalent.

[B24-entropy-22-01273] 24.$C_{<}^{↑}$ is undefined here.

[B25-entropy-22-01273] 25.Notice that ∑*c_j_* = *m* + *n* − ∑*c_ℓ_*.

[B26-entropy-22-01273] 26.The claim (i) of Proposition 3 is likely to be known in the context of majorization, but we did not find a suitable reference.

[B27-entropy-22-01273] 27.What is considered “equivalent” is dictated by the physics involved, rather than the concept of the effective number itself. For example, it is possible to encounter a situation where the dimensions of “usefully equivalent” subspaces are not the same.

[B28-entropy-22-01273] 28.The meaning of {∣*i*^(*k*)^〉} ≡ {∣*i*^(*k*)^〉∣*i* = 1,2,…,*N_k_*} targeting {∣*i*〉} depends on the context, but is usually clear on physics grounds.

[29] Leinster T., Cobbold C.A. (2012). Measuring diversity: The importance of species similarity. Ecology.

[30] Leinster T., Meckes M.W. (2016). Maximizing Diversity in Biology and Beyond. Entropy.

[31] Arnold B. (1987). Majorization and the Lorenz Order: A Brief Introduction.

[32] Marshall A.W., Olkin I., Arnold B.C. (2011). Inequalities: Theory of Majorization and Its Applications.

